# A Feasibility and Pilot Study of a Personalized Nutrition Intervention in Mobile Food Pantry Users in Northeastern Connecticut

**DOI:** 10.3390/nu13092939

**Published:** 2021-08-25

**Authors:** Dalia Marmash, Kyungho Ha, Junichi R. Sakaki, Rachel Hair, Emma Morales, Valerie B. Duffy, Michael Puglisi, Ock K. Chun

**Affiliations:** 1 Department of Nutritional Sciences, University of Connecticut, Storrs, CT 06269, USA; dalia.marmash@uconn.edu (D.M.); junichi.sakaki@uconn.edu (J.R.S.); rachel.hair@uconn.edu (R.H.); Emma.morales@uconn.edu (E.M.); michael.puglisi@uconn.edu (M.P.); 2 Department of Food Science and Nutrition, Jeju National University, Jeju 63243, Korea; kyungho.ha@jejunu.ac.kr; 3 Department of Allied Health Sciences, University of Connecticut, Storrs, CT 06269, USA; valerie.duffy@uconn.edu

**Keywords:** mobile food pantry, personalized nutrition, nutrition education, feasibility study, pilot study

## Abstract

Objective: This pilot study assessed the effectiveness and acceptability of personalized nutrition intervention for mobile food pantry users. Methods: The 8-week intervention recruited 25 participants in the control (*n* = 13) and in the treatment (*n* = 12) groups (60% obese). Personalized nutrition and health reports were generated based on baseline dietary intake and health status. The treatment group received weekly phone counseling and nutrition education, while the control group was only contacted to ensure compliance. The primary outcomes were 8-week changes in weight and diet quality score, assessed by the Healthy Eating Index. Results: The acceptability of the intervention was assessed by the eligibility rate, recruitment rate (62.5%), and drop-out rate (36%). Following the intervention, there was a significant decrease in weight (mean ± standard deviation, −2.3% ± 2.4%) among all participants (*p* < 0.05). Diet-quality improved (4.54% in treatment vs. 0.18% in control), but was ultimately non-significant (*p* = 0.284). Conclusions and Implications: A personalized nutrition education intervention in mobile food pantry users may be an acceptable and effective intervention to encourage weight loss through dietary improvements.

## 1. Introduction

Low-income, food-insecure individuals have a great risk of low diet quality, nutrient inadequacy, and poor health outcomes [1,2,3]. More specifically, fresh produce, dairy, and whole grains are usually under-consumed, while saturated fats, refined grains, added sugar, and sodium are consumed in excess [1,3,4,5]. While these dietary disparities are largely attributed to lack of financial resources [6,7,8,9,10], the diet and health of low-income individuals can improve with increased access to fresh produce in conjunction with well-designed nutrition interventions [11,12,13,14].

Efforts to improve the diet quality of low-income individuals most commonly focus on improving healthy food availability, increasing nutrition and health education, and providing individuals with the tools to prepare healthier meals at home [12]. Many of these interventions are implemented at food assistance sites or among specific program users [12,14,15]. Unfortunately, there are many barriers associated with these interventions, including low participation rates, limited trained staff, and scarce resources [11,12]. It is crucial to consider these barriers when developing a nutrition intervention, allowing for the adaptation of protocols to optimize efficacy [16]. The coupling of food distribution and nutrition education intervention studies may enhance participation in these interventions and can provide resources to individuals with the highest need [17].

Personalizing nutrition education messages to a variety of the individual’s characteristics can be even more successful in eliciting long-term behavior and dietary change [18,19,20]. One randomized trial found that personalized messaging to the individual’s dietary intake was more effective than general messaging in improving diet quality, requiring only dietary and anthropometric data [19]. In fact, using more personal or invasive data such as genotype was not more effective than using dietary/anthropometric data alone, indicating that “personalizing” the intervention program may be the key to the success of nutrition education. Of interest is if personalized nutrition education can be incorporated into an intervention for mobile food pantry clients and if this intervention is acceptable and useful to change diet and health. Many SNAP-Ed programs provide direct, individualized recommendations to their clients, with promising results on dietary behavior changes and participant self-efficacy [21]. Mobile food pantry users have not been widely studied, and an assessment of their specific barriers to healthy eating, as well as the most acceptable methods of introducing nutrition education, is necessary.

Prior to the development of a large-scale intervention in a novel population, conducting a pilot study is often advised [16]. By helping to identify potential barriers researchers might face, they allow for appropriate changes to be made prior to a large-scale roll-out. In order to truly assess the feasibility of the larger study, similar methods are used in the pilot study. The acceptability of study protocols can also be assessed using post-intervention questionnaires [16]. Thus, feasibility studies are an important contribution to the literature and allow for the strengthening of future interventions within the study population.

While personalized nutrition education has been demonstrated to be successful, evidence is lacking for diverse, low-income food-insecure populations. Therefore, the aims of this pilot study were to assess the acceptability and short-term effectiveness of a personalized nutrition education intervention for mobile food pantry users in an ethnically diverse environment.

## 2. Materials and Methods

### 2.1. Study Design

Baseline interviews were conducted in Spring 2020 (pre-COVID-19). Participants were recruited from two mobile food pantry sites in Willimantic, Windham County, Connecticut. Inclusion criteria required participants to be at least 19 years of age and to have visited a food pantry at least once prior to recruitment. Consent forms were signed prior to study commencement. The study protocol was approved by the BLINDED Institutional Review Board (#H19-206).

Researchers conducted three surveys at baseline. The first collected sociodemographic, lifestyle, and health-related data. Next, the United States Department of Agriculture (USDA) Food Security Questionnaire and the National Cancer Institute (NCI) Dietary Screener Questionnaire (DSQ) were administered. Participants were asked to complete a 3-day dietary record on three non-consecutive days and to return it to the study center upon completion. In total, 83 participants completed the first day of interviews, and 40 submitted their 3-day dietary record. A more detailed description of the sociodemographic, dietary, and health-related characteristics of the study population has been published elsewhere [22]. Among the 40 participants who completed baseline surveys, anthropometric measures, and a dietary record, 25 participants were interested in participating in the intervention study. Participants were randomized into the control (*n* = 13) or treatment (*n* = 12) group using excel.

Research staff were assigned participants to follow up with throughout the 8-week study. Three staff members were responsible for direct participant contact, and they were trained by a registered dietitian prior to study commencement. Weekly team meetings were also conducted to ensure staff were trained on the topics for the following week and to identify the specific points to be covered during each participant interaction.

Additionally, participants in both the control and treatment groups received a baseline instructional call with trained study personnel to explain the intervention study flow, the materials included and to answer any questions. Personalized nutrition and health reports were reviewed in detail to support participant understanding. This call lasted approximately 20 min.

Those in the treatment group were contacted at baseline and weekly throughout the intervention. Each week, participants were sent nutrition education fliers in the mail on nutrition-related topics to review during 10-min weekly phone meetings with study personnel. These topics included tips on limiting sodium, added sugar, and saturated fat, portion size, and nutrition label information, and tips for buying healthy food on a budget. Study personnel also encouraged participants to follow their personalized nutrition-related goals each week and reminded them to fill out their progress logs. At week 8, exit interviews were conducted, and participants were instructed to fill out their 3-day dietary records.

Participants in the control group were contacted at baseline, week 4, and week 8. They did not receive weekly nutrition education fliers or weekly follow-ups with research staff. At week 4, study personnel ensured they had been recording the required information in their progress log. At week 8, participants were given an exit interview and were instructed to fill out their 3-day dietary records. Following the completion of their records, they were instructed to submit their forms to the study center.

Following the 8-week intervention, 10 participants returned their completed materials to the study center, completing the trial. Following the first day of baseline interviews, a $20 Walmart gift card was awarded, and an additional $10 was given to those who returned their completed 3-day food records. Following the completion of the intervention study, another $20 Walmart gift card was awarded.

### 2.2. Development of the Intervention

Initially, the intervention was designed to provide nutrition education through in-person meetings at the mobile food pantry distribution sites to encourage participants to choose and prepare healthier foods. However, due to the COVID-19 pandemic, it was no longer possible to meet participants at mobile food pantry sites, so the intervention shifted to fully remote, using phone calls to speak with participants. The intent of the Easy! Nudges intervention was to provide personalized yet simple and feasible nutrition education guidelines to the participants. The intervention was conducted solely using phone or mail communications. The literature supports the use of personalized nutrition messaging and attainable goal setting as an effective strategy for behavior change [18,19,20]. Components of the Social Cognitive Theory, the Health Belief Model, and the Transtheoretical Model of Behavior Change provided a theoretical framework for this study. Personalized nutrition reports were generated based on each participant’s anthropometric measurements, health status, and dietary data at baseline. In addition, three diet-related goals were selected by the research staff for each participant based on their baseline dietary intake. Each participant was encouraged to complete a weekly progress log for self-monitoring to help them keep track of their own weight and waist circumference changes, physical activities, and goal adherence. General nutrition reports with information on healthy eating and physical activity and healthy, affordable recipes also were provided.

Materials were generated to be as clear and easily understood as possible. Goals were attainable and thoroughly explained during the baseline intervention interview. Trained staff clarified any confusion or questions participants voiced. Participants were also instructed to call or email research staff with any questions or concerns.

The study population was largely Hispanic or Latino, and many participants were more confident communicating in Spanish. Thus, these participants’ study materials were translated into Spanish, and they were assigned fluent Spanish-speaking study personnel to conduct all interviews. We also selected culturally appropriate, healthy, and affordable recipes to distribute to all participants. This supported that all participants were comfortable, and that they fully understood all of the provided materials.

### 2.3. Anthropometric Measurements

At baseline, technicians measured the participants’ height, weight, and waist circumference in a private area. Jackets and shoes were removed, and weight was measured using a digital scale. Waist circumference was measured using a measuring tape directly above the ileac crest, with bulky clothing removed. Height was measured without shoes using a stadiometer.

During the intervention, participants were given a self-balancing digital scale and were asked to weigh themselves each week in the morning, wearing minimal clothing on a hard surface. Participants were also instructed to measure their waist circumference directly above the ileac crest using the provided measuring tape. Pictures demonstrating proper measurement technique were given to participants to limit confusion.

### 2.4. Physical Activity

Participants were asked to record their average weekly physical activity throughout the intervention. They recorded the number of days per week and the average number of minutes per day but did not record activity type.

### 2.5. Recommendation Adherence

Participants were each given 3 tailored goals to follow throughout the intervention. Goals included limiting sodium, saturated fat, and added sugar intake, increasing fruit, vegetable, whole grain, and low-fat dairy intake, and moderating portion sizes. These goals were chosen for participants by research staff due to their impact on total diet quality and health outcomes. For each participant, goals were selected by research staff based on their baseline dietary behaviors and health status. Each week, participants recorded whether or not they completed this goal on at least one day during the week. Then, technicians translated the recommendation adherence into a numerical score, with 0 indicating no adherence and 3 indicating adherence to all three goals. Research staff were instructed by the registered dietitians to adhere to the Nutrition Care Process throughout the intervention by helping participants set realistic goals, providing appropriate education materials for the population, and by monitoring the participants on a regular basis.

### 2.6. Dietary Intake

Baseline dietary intake included data from the DSQ and completed 3-day dietary records. Participants then completed a second 3-day dietary record following the 8-week intervention. The 3-day dietary records were completed on non-consecutive days, two weekdays, and one weekend day. Dietary data was input into the Nutrition Data System for Research (NDSR) 2020 version (University of Minnesota, Minneapolis, MN, USA).

### 2.7. Acceptability Tests

Exit surveys were administered to each participant following the 8-week intervention. Questions regarding study acceptability and barriers to study completion were collected. Feasibility was assessed by recruitment rate (number randomized/number completing baseline survey and 3-day food record) and eligibility rate (number eligible/number completing baseline survey). Withdrawal rate (number of withdrawals/number randomized) was also used to assess acceptability, in addition to participants’ exit survey data [16].

### 2.8. Data Analysis

Values are presented as mean ± standard deviation or as *n* (%). All tests performed were non-parametric. Differences in anthropometric, dietary, and physical activity measures in the total sample were measured using Wilcoxon signed-rank tests, and differences between the control and treatment group were measured using Wilcoxon rank-sum tests. All *p* values were two-sided, and *p* < 0.05 was considered statistically significant. Data analysis was conducted using SAS software (version 9.4; SAS Institute, Cary, NC, USA).

## 3. Results

The aim of the current study was to assess the changes in dietary intake pre- and post-intervention. Changes in food group intake provided insight into the changes in dietary quality. Due to the nature of this study, specific micro- and macro-nutrient data were not included but could be helpful in future, more comprehensive studies.

Among 40 participants who returned their 3-day dietary records to the study center during pre-intervention, 25 showed interested in participating in the intervention study and were randomized into the control (*n* = 13) and treatment (*n* = 12) groups. This translated to a 62.5% recruitment rate and a 48.2% eligibility rate. Three participants withdrew before baseline interviews, and six withdrew at week 4. Withdrawal was due to health incidents (*n* = 3), family emergencies (*n* = 2), and a change in phone number resulting in lost contact (*n* = 4). Sixteen completed the 8 weeks of counseling (64%), and 10 participants sent in their materials and completed a second 3-day dietary record (40%). Thus, 10 participants completed the entire intervention study, including the submission of all related paperwork.

Baseline data of the 10 participants are included in Table 1. Overall, participants were largely female (90%), overweight or obese (BMI 30.8 ± 7.8 kg/m^2^), and had low intakes of fruits, vegetables, and whole grains. Given that the participants’ average BMI was above 30 kg/m^2^ and waist circumference indicated central obesity, it is likely that a majority of the participants had excess adiposity. There were no significant differences in baseline anthropometric or dietary intake between the control and treatment groups.

Table 2 shows dietary intake changes from baseline following the 8-week intervention. Healthy Eating Index (HEI) scores had a greater improvement in the treatment group compared to the control group following the intervention (4.54% vs. 0.18%), although the difference was not statistically significant.

**Figure 1:**
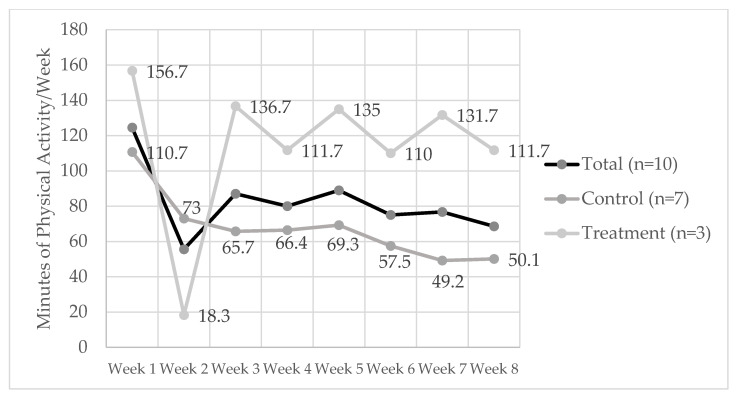
Physical activity (minutes/week) in study participants (*n* = 10) over 8 weeks.

Figure 1 shows the average total weekly physical activity of participants. Although differences did not reach statistical significance, trends indicated a higher activity level in the treatment group compared to the control group. Overall, weekly time remained relatively stable throughout the intervention.

**Figure 2:**
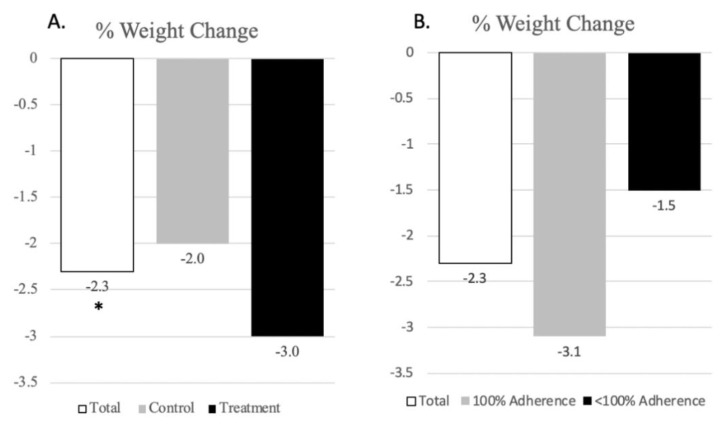
Mean changes in weight (% change) in total participants (*n* = 10), the control (*n* = 7) and treatment (*n* = 3) groups (**A**) and by recommendation adherence (**B**). * indicates statistical significance using Wilcoxon signed rank test (*p* < 0.05).

Figure 2 shows changes in anthropometric measures following the 8-week intervention (*n* = 10). In all participants, there was an average weight loss of 4.2 pounds (*p* < 0.05). Weight loss was greater in the treatment group compared to the intervention group (−3.0% vs. −2.0%), although differences did not reach statistical significance due to the small sample size. Figure 2 also shows weight change by recommendation adherence. Those who adhered to their dietary goals throughout the eight weeks lost more weight than those who did not (−3.1% vs. −1.5%), although it was not statistically significant. Changes in waist circumference were minimal due to the short follow-up time.

## 4. Discussion

This personalized nutrition education intervention assessed the acceptability and utility of tailored interventions in mobile food pantry users. Participant interest was high, as indicated by participant recruitment rates; however, adverse health events and unreliable contact information were significant barriers to program completion. Overall, all participants had significantly reduced weight and percent weight change following the 8-week intervention. Furthermore, the intervention group lost more weight, participated in more physical activity, and had greater goal adherence than the control group, although differences were not statistically significant. Diet quality changes were insignificant, but overall trends suggest improvements compared to baseline.

Feasibility studies are useful tools, allowing for the assessment of intervention methods prior to a large-scale study. The current intervention was conducted during the COVID-19 pandemic, requiring study communications to occur via mail or phone. This posed a significant challenge during recruitment, as many participants did not have stable addresses or phone numbers. Furthermore, the majority of participant withdrawals could be attributed to significant health events or family emergencies. Despite these barriers, 64% of recruited participants completed the 8-week counseling, and 40% sent in their completed materials. Compared to similar intervention studies, these measures of feasibility indicate a well-received intervention [24].

Although the main aim of the current study was to assess the feasibility and efficacy of the intervention methods, outcome measures were recorded for preliminary insight into study efficacy. Many past nutrition interventions have not observed large, sustainable changes in diet quality, most likely due to the variety of factors impacting diet quality in this low-income, underserved population [12]. Modest improvements in fruit and vegetable intake have been achieved following an education-based intervention, however [25,26,27]. The trends of the current data suggest a slight improvement in diet quality, which may be better observed with a larger sample size. Furthermore, there was a 4.54% improvement in diet quality in the treatment group compared to a 0.18% improvement in the control group, which despite being statistically insignificant, suggests that increased contact with study staff may be effective at eliciting more meaningful behavior change. One possible explanation for this insignificant diet quality improvement is the decreased mobile food pantry distribution throughout the COVID-19 pandemic. Baseline data were recorded prior to the pandemic when community aide organizations were operating on a more regular schedule. Throughout the pandemic, many mobile food pantries decreased distribution at smaller community sites in favor of larger-scale distribution centers. This left many participants with no car without a reliable, biweekly source of fresh produce and meats.

Despite the small sample, weight loss was significant following the intervention in all participants. Weight loss was also greater in the treatment group and in those who adhered to their nutrition goals more often, although these trends did not reach significance. This suggests weight loss was impacted by the frequency of contact with trained research staff and by participant motivation in following the recommendations provided. These preliminary trends are supported by behavior change models, which promote encouraging participant self-efficacy and encourage the development of a support network to increase participant goal achievements [20].

A future, larger-scale intervention can utilize these insights to bolster study efficacy. A high participant attrition rate should be considered and factored into sample size calculations. Additionally, obtaining as many forms of contact as possible will help limit the number of participants lost to disconnected phone numbers and changed addresses. Future studies in this participant population may also benefit from developing a partnership with a community organization, such as a local health clinic, where the staff have established connections with the study participants. This would limit participant drop-out due to changes in phone number and may help elevate participant interest. Increasing the study duration might also yield more significant results and will allow for the assessment of longer-term behavior change. In the current study, certain outcome measures, such as waist circumference, saw little change from baseline. A longer-term intervention may allow for the observation of more significant changes in anthropometric and diet quality measures.

Due to the nature of this feasibility study, there are several limitations to consider when interpreting the data. First, the sample size is small, with only 10 total participants included in the analysis. This led to less generalizable but more conservative results. Since the main aim of this study was to assess the feasibility and acceptability of the methodology, measuring the trends in outcome changes is an adequate preliminary measure of study efficacy [26]. Another limitation is the short follow-up time of 8-weeks. Future interventions will have an increased follow-up time to increase the power of the findings. Finally, exercise type or intensity was not assessed. Although not quantified, discussions with research staff indicate that most exercise was low intensity for participants. While low intensity physical activity results in significant health benefits, future studies may focus on ensuring that moderate- to vigorous-intensity exercise is recommended and quantified.

The current study provides insight into the implementation and outcomes of an acceptability study using personalized nutrition intervention in this unique population. Future interventions should keep in mind the barriers to executing a study in this population to ensure optimal use of time and resources. Partnership with community organizations could reduce participant drop-out due to changes in contact information, increasing study completion rates. Data suggest the personalization of nutrition education is a promising method of improving diet quality and health status in mobile pantry users. Adoption of these personalized nutrition education techniques may yield more significant, sustained behavior change results in low-income populations.

## Figures and Tables

**Table 1:** Baseline characteristics of study participants (*n* = 10).

Variable	Total (*n* = 10)	Control (*n* = 7)	Treatment (*n* = 3)	*p*-Value ^1^
Male/Female	1/9	1/6	0/3	
Anthropometric				
Age (years)	54.6 ±12.4	52.9 ± 14.5	58.7 ± 5.7	0.657
Height (m)	1.57 ± 0.05	1.59 ± 0.04	1.54 ± 0.04	0.205
Weight (kg)	76.3 ± 20.7	77.6 ± 25.2	73.3 ± 2.6	0.825
BMI (kg/m^2^)	30.8 ± 7.8	30.7 ± 9.5	31.0 ± 0.6	1.000
Waist circumference (cm)	102.1 ± 10.6	100.9 ± 12.7	105.0 ± 1.5	
Clinical				
Diabetes (%)	30.0	28.6	33.0	1.000
Hypertension (%)	40.0	28.6	66.0	1.000
Obesity (%)	60.0	43.0	100.0	1.000
HEI scores ^2^				
Total Score (100)	55.9 ± 10.5	56.6 ± 12.2	54.2 ± 6.7	1.000
Total Fruits (5)	3.1 ± 1.3	2.8 ± 1.5	3.7 ± 0.4	0.511
Whole Fruits (5)	3.3 ± 1.6	3.0 ± 1.8	3.9 ± 1.0	0.505
Total Vegetables (5)	3.4 ± 1.6	3.2 ± 1.8	3.9 ± 1.0	0.825
Greens and Beans (5)	2.1 ± 2.0	2.5 ± 2.0	1.1 ± 1.9	0.366
Whole Grains (10)	2.4 ± 2.0	2.3 ± 2.1	2.5 ± 2.2	0.823
Dairy (10)	4.8 ± 3.3	6.1 ± 3.1	1.2 ± 1.8	0.145
Total Protein Foods (5)	4.5 ± 1.0	4.8 ± 0.6	3.8 ± 1.6	0.199
Seafood and Plant Proteins (5)	2.6 ± 1.7	3.0 ± 1.7	1.7 ± 1.7	0.315
Fatty Acids (10)	5.0 ± 2.5	4.4 ± 2.1	6.4 ± 3.1	0.284
Refined Grains (10)	6.0 ± 2.4	6.2 ± 2.8	5.5 ± 1.5	1.000
Sodium (10)	3.2 ± 2.2	2.6 ± 2.0	4.7 ± 2.1	0.284
Added Sugar (10)	9.4 ± 1.1	9.7 ± 0.5	8.8 ± 1.9	0.484
Saturated Fat (10)	6.1 ± 2.3	6.1 ± 2.4	6.3 ± 2.7	1.000
Energy	1580.8 ± 683.8	1692 ± 797.6	1319.5 ± 221.4	0.659

All values are mean ± standard error or (%). ^1^ Measured using Wilcoxon rank-sum test. ^2^ Maximum possible score is indicated for each dietary component, with a higher score indicating greater adherence to the Dietary Guidelines 2015–2020 [23].

**Table 2:** Changes in dietary quality assessed by Healthy Eating Index scores between baseline and 8 weeks (*n* = 10).

	Change Following 8-Week Intervention				
Variable	Change from Baseline for Total Participants (*n* = 10)	*p*-Value ^1^	Change from Baseline in Control (*n* = 7)	Change from Baseline in Treatment (*n* = 3)	*p*-Value ^2^
HEI Scores ^3^					
Total Score (100)	1.49 ± 7.10	0.770	0.18 ± 7.09	4.54 ± 7.47	0.284
Total Fruits (5)	0.13 ± 0.83	0.652	0.02 ± 0.98	0.38 ± 0.33	0.386
Whole Fruits (5)	−0.19 ± 1.68	0.676	−0.29 ± 1.85	0.04 ± 1.53	0.825
Total Vegetables (5)	0.17 ± 1.77	0.922	0.47 ± 1.76	−0.52 ± 1.95	0.659
Greens and Beans (5)	−0.37 ± 1.94	0.469	−0.77 ± 1.70	0.56 ± 2.55	0.439
Whole Grains (10)	1.05 ± 2.53	0.232	1.33 ± 2.40	0.40 ± 3.26	0.825
Dairy (10)	−0.66 ± 4.23	0.695	−1.51 ± 3.87	1.33 ± 5.22	0.511
Total Protein Foods (5)	−0.37 ± 1.38	0.461	−0.83 ± 1.38	0.68 ± 0.70	0.171
Seafood and Plant Proteins (5)	0.14 ± 1.84	0.922	−0.04 ± 1.93	0.55 ± 1.92	0.736
Fatty Acids (10)	0.72 ± 3.61	0.846	1.05 ± 3.65	−0.04 ± 4.16	0.511
Refined Grains (10)	−0.57 ± 3.46	0.734	−0.79 ± 4.09	−0.03 ± 1.74	0.825
Sodium (10)	1.65 ± 3.52	0.131	2.06 ± 4.20	0.69 ± 0.97	0.511
Added Sugar (10)	−0.97 ± 1.39	0.074	−1.19 ± 1.54	−0.44 ± 1.00	0.511
Saturated Fat (10)	0.76 ± 3.67	0.492	0.66 ± 3.50	0.97 ± 4.88	0.659
Energy	−208.73 ± 795.10	0.432	−21.22 ± 939.83	−184.23 ± 440.10	1.000

All values are mean ± standard error. ^1^ Measured using Wilcoxon signed-rank test. ^2^ Measured using Wilcoxon rank-sum test. ^3^ Maximum possible score is indicated for each dietary component, with a higher score indicating greater adherence to the Dietary Guidelines 2015–2020 [23].

## Data Availability

The data presented in this study are available on request from the corresponding author.
